# Interplay of rice vitamin E under osmotic and extreme temperature stresses revealed by a comparative transcriptomic approach

**DOI:** 10.1186/s12870-025-07374-0

**Published:** 2025-10-03

**Authors:** Sara Kazemzadeh, Naser Farrokhi, Asadollah Ahmadikhah, Pär K. Ingvarsson

**Affiliations:** 1 Department of Cell and Molecular Biology, Faculty of Life Sciences & Biotechnology, Shahid Beheshti University, Tehran, Iran; 2 Department of Plant Biology, Swedish University of Agricultural Sciences, Uppsala, Sweden

**Keywords:** Cold stress, Meta-analysis, *Oryza sativa*, Tocopherol, Transcription factor

## Abstract

**Background:**

Rice (*Oryza sativa* L.) is vulnerable to abiotic challenges. Understanding stress response mechanisms is therefore a priority for enhancing rice development. Tocopherol is a known antioxidant that helps plants adapt to various abiotic stresses. We analysed bibliographic data from 13 years of studies on abiotic stresses. We also performed a meta-analysis of 231 microarray samples from 12 different studies on genotypes sensitive and tolerant to drought, salinity, heat, and heat-related effects on vitamin E biosynthesis in rice. Common differentially expressed genes (DEGs − 30) were identified with *p*-value < 0.05 and |log_2_FC| > 1. An in silico expression analysis of the DEGs and a Protein-Protein Interaction (PPI) network analysis were performed using bioinformatics tools.

**Results:**

Our findings showed that 13 structural Genes and 17 transcription factors, including *OsGGPPS1*, isochorismatase hydrolase, aminotransferase, *OsVTE3*, shikimate kinase, and the families of bHLH, WRKY, bZIP, and C2H2 transcription factors, are all involved in vitamin E biosynthesis under drought, cold, and heat stresses in rice. *OsWRKY77* was commonly expressed in both cold and heat-sensitive genotypes, and in aminotransferase between drought and cold in tolerant genotypes.

**Conclusions:**

The analysis showed that abiotic stresses, except for salt stress, induce genes involved in vitamin E biosynthesis. Cold stress induced more intense molecular responses compared to other types of stress. Our results can provide insight into the regulatory mechanisms involved in response to selected abiotic stresses, which ultimately can contribute to the development of stress-resistant or tolerant rice cultivars.

**Graphical Abstract:**

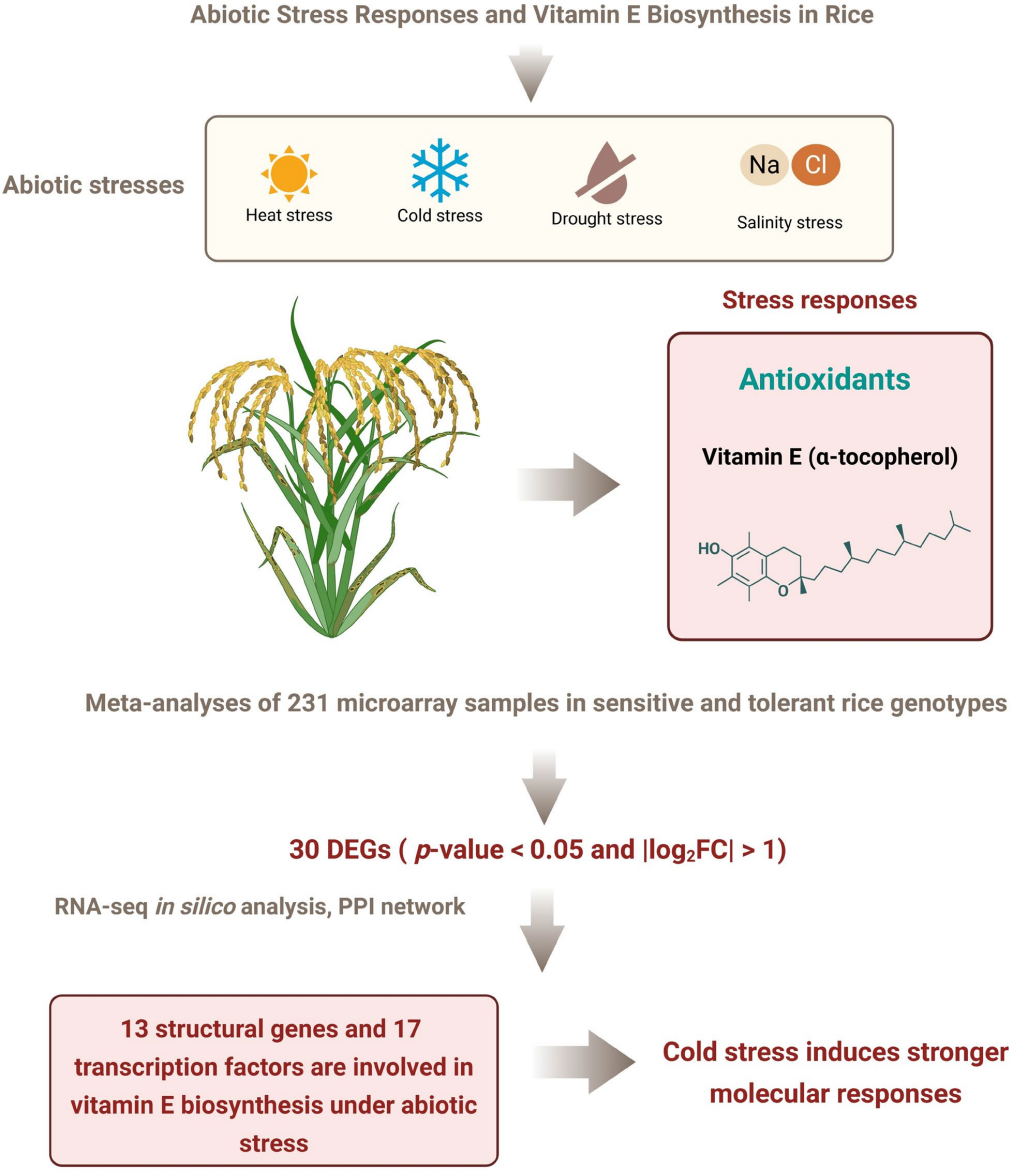

**Supplementary Information:**

The online version contains supplementary material available at 10.1186/s12870-025-07374-0.

## Background

Rice (*Oryza sativa*) is a staple food for more than half of the world’s population, and it is currently cultivated across approximately 162 million hectares of land, with an annual global production of about 756 million tons [1]. Various abiotic stresses can reduce rice production, resulting in a yield loss of approximately 30% of the annual production [2]. Among the most serious abiotic stresses experienced by crops such as rice are drought and salinity, as they alter the ionic and osmotic equilibria of the cell [3]. Rice is also susceptible to temperature stresses, particularly cold stress at the booting stage and heat stress at the flowering stage [4].

While these stresses are often studied individually, in nature, plants frequently face simultaneous or sequential stresses, which increasingly occur in combinations, mainly due to climate change. To capture this complexity, the concept of multifactorial stress combination (MFSC) has been introduced, referring to the simultaneous or sequential impact of three or more abiotic and/or biotic stresses in plants. Combined abiotic stresses threaten food security by impairing plant tolerance-related mechanisms, i.e., growth and yields [5, 6]. Recent studies emphasise the importance of integrated multi-omics (panomics) and AI-driven approaches to achieve a holistic understanding of plant responses under MFSC. For instance, a recent study confirmed that five low-level combined stresses (salinity, heat, herbicide paraquat, phosphorus deficiency, and cadmium) significantly reduced the growth and biomass of rice (*Oryza sativa* L.) and maize (*Zea mays* L.), suggesting that the severity of combined stresses may increase with their number [7]. These insights underline the urgent need for modern stress biology to move beyond single-stress frameworks and focus instead on the mechanisms of plant acclimation to complex environments [5].

Plant responses to stress are complex, involving a host of physiological, biochemical and molecular changes [8, 9]. After experiencing drought stress, numerous rice genes have been reported to be differentially expressed, with ~ 5,000 genes upregulated and ~ 6,000 downregulated [10, 11]. For example, *DRO1* induces root elongation and deep rooting in transgenic rice [12]. Genes such as *OsPYL/RCAR5* and *EcNAC67* delay leaf rolling and induce higher root and shoot mass in rice in response to low water availability [13, 14]. *WRKY* genes play several essential roles in plant development, for example, in response to drought stress [15]. Several DREBs have been identified in rice based on genome-wide conserved sequence analyses, some of which are also responsive to salinity stress. *AREB/ABF* is a bZIP (basic leucine zipper) transcription factor that binds to *cis*-acting elements responsive to ABA [16], and many *AREB*/*ABF*-homologous genes have also been implicated in plant responses to salt stress. *OsNAC* might play a crucial role in the crosstalk between different types of stress signalling genes via up-regulating stress-responsive genes and thus enhancing salt tolerance [17]. So far, 97 genes related to heat tolerance in rice have been identified. For example, overexpression of *OgTT1* in rice could significantly improve heat tolerance. *SNAC3* encodes a stress-responsive NAC transcription factor. It balances H_2_O_2_ by regulating the expression of the ROS genes, thus achieving heat tolerance [18]. *OsCOLD1* contributes to cold tolerance in rice by encoding a plasma-membrane or endoplasmic reticulum (ER) located protein that regulates G-protein signalling [19].

Plants have developed several antioxidant defence systems to cope with oxidative stress, with vitamin E being an important nonenzymatic antioxidant. Vitamin E, including tocopherols and tocotrienols. Vitamin E belongs to a group of amphiphilic lipids present in plant plastids, which are exclusively synthesised by photosynthetic organisms, acting as antioxidants [20, 21]. Vitamin E, first described as α- and β-tocopherols, was isolated from wheat (*Triticum aestivum* L.) seed oil, while γ- and δ-tocopherols were extracted from oilseeds [22]. Tocopherols play vital roles in plant adaptation to stress conditions such as drought [23], salinity [24], extreme temperature [25], radiation [26], and toxic metals [27]. For example, one α-tocopherol molecule can efficiently counteract about 120 singlet oxygen species before being eliminated [28]. External application of α-tocopherol to plants under stress conditions has improved chlorophyll content and photosynthesis rates [29–31].

Identifying and understanding the mechanisms plants employ in response to abiotic stress would improve plant production [32], especially under stress conditions. To gain deeper insight into the complex molecular systems and to identify the key pathways and mechanisms involved in cellular responses to abiotic stresses, the use of statistical and computational approaches is essential. Advances in high-throughput technologies, such as microarray and RNA-Seq, have enabled the simultaneous analysis of thousands of genes across diverse conditions.

Although plants frequently encounter multiple stresses in nature, the aim of this study was to investigate the expression of genes affecting the vitamin E biosynthesis pathway in rice grain under selected abiotic stresses, including osmotic and temperature stresses, in susceptible and resistant rice genotypes using an *in*-*silico* comparative transcriptomics approach. The central biological question we ask is how genes associated with vitamin E biosynthesis respond to osmotic and temperature stresses. While transcriptomics provides valuable insights, future work incorporating multi-omics approaches will be needed to understand responses to multifactorial stress combinations.

## Methods

### Data collection

Genes known to influence the genetic control of vitamin E content in rice seeds were selected based on our recent GWAS analyses [33, 34] and others [25, 35, 36] (Supplementary Table S1). Publicly available microarray datasets were retrieved from the NCBI Gene Expression Omnibus (GEO) under platform GPL2025, which contained 136 probe sets corresponding to vitamin E-related genes (Supplementary Table S2). The expression dataset included a total of 231 samples from rice seedlings, leaves and Flag leaves of sensitive and tolerant genotypes exposed to drought, salinity, cold, and heat stress conditions (Table 1). Raw data were preprocessed using standard bioinformatics workflows. The data were consolidated, and comparability was ensured between the stress and control samples. Batch effects were corrected using the ComBat function from the SVAR package. Probes lacking gene annotations were excluded, and for genes with multiple probe sets, expression values were averaged. The gprofiler Gene ID Conversion Tool (g: convert) was used to convert Affymetrix IDs (AFFY_RICE) to EntrezGene and to annotate the functions of genes associated with vitamin E biosynthesis (https://biit.cs.ut.ee/gprofiler/convert*).* The underlying annotation database was based on the *Oryza sativa* Japonica Group genome.

**Table 1:** Sensitive and tolerant rice genotypes used for microarray experiments

Accession	Treatment	Line	Line Details	Reference	Tissue type
GSE21651	Drought/Cold	IR64	sensitive	Mishra et al. 2018	Seedlings
GSE24048	Drought	Azucena	tolerant	Price et al. 2017	Leaves
GSE26280	Drought	DK151	tolerant	Wang et al. 2011	Leaves
GSE25176	Drought	IRAT109	tolerant	Ding et al. 2013	Flag leaves
GSE6901	Drought/Cold	IR64	sensitive	Jain et al. 2007	Seedlings
GSE41647	Drought	Dagad deshi	tolerant	Borah et al. 2017	Seedlings
GSE16108	Salt	MI48	sensitive	Pandit et al. 2010	Seedlings
GSE41650	Salt	Nonabokra	tolerant	Borah et al. 2021	Seedlings
GSE38023	Cold	Li-Jiang-Xin-Tuan-Hei-Gu	tolerant	Zhang et al. 2012	Seedlings
GSE37940	Cold	K354	tolerant	Zhang et al. 2012	Seedlings
GSE14275	Heat	Zhonghua 11	sensitive	Hu et al. 2009	Seedlings
GSE41648	Heat	Annapurna	tolerant	Borah et al. 2021	Seedlings

Differential expression analysis was conducted using limma and edgeR packages in R. To control for false positives due to multiple testing, Benjamini–Hochberg false discovery rate (FDR) correction was applied, and genes with an adjusted *p*-value < 0.05 and |log₂FC| > 1 were considered differentially expressed. Heatmaps were generated using the ggplot2 package in R, with a red/green colour scale where red indicates upregulation and green indicates downregulation. Additionally, Venn diagrams were generated using limma to identify overlapping DEGs across different stress conditions.

### In Silico RNA-seq expression analysis of DEGs and functional enrichment analysis

Evidence of gene expression of DEGs under cold, heat, salinity, drought stresses and in different tissues of rice, including seedling, leaves and shoots, was checked via available RNA-Seq data available in the Rice Genome Annotation Project (RGAP, https://rice.uga.edu/*)* and the Plant Public RNA-seq Database (https://plantrnadb.com/ricerna/*).* A red/blue colour scheme was used, where “red” represents up-regulation and “blue” represents down-regulation of respective genes. To map transcriptomic data, we employed the MapMan software (ver. 3.6.0RC1). Osa_RAPDB_ mapping files were downloaded from the Map-Man store server.

(https://mapman.gabipd.org/web/guest/mapmanstore*).*

### Protein–protein interaction network

To construct protein association networks between DEGs involved in tocopherol biosynthesis, we used STRING (Search Tool for the Retrieval of Interacting Genes, https://string-db.org/*)* with the confidence parameter threshold set to 0.40, the maximum number of interactors in the first shell Limited to 10, and with none in the second shell. To analyse the network model for putative interacting partners, we used query sequences consisting of the amino acid sequences of relevant proteins. To visualise the network, we use Cytoscape v3.9.1.

### Gene ontology (GO) enrichment and KEGG pathway determination

To better understand the function of DEGs in the cell, we classified DEGs using ShinyGO v0.75 (http://bioinformatics.sdstate.edu/go/). The GO database generates an overview of the functional classification from a gene ID list. This functional classification can then be divided into biological processes, molecular functions, and cellular components. Go analyses were performed using ShinyGO, a web-based tool for exploring GO term enrichment in genomic datasets. The results can be visualised in an interactive and user-friendly way, making it straightforward to identify functional categories that are overrepresented in the data. We used the online version of the ShinyGO software for our GO and KEGG enrichment analysis of DEGs [37].

## Results

### Identification of the stress-responsive core DEGs in rice

**Fig. 1:**
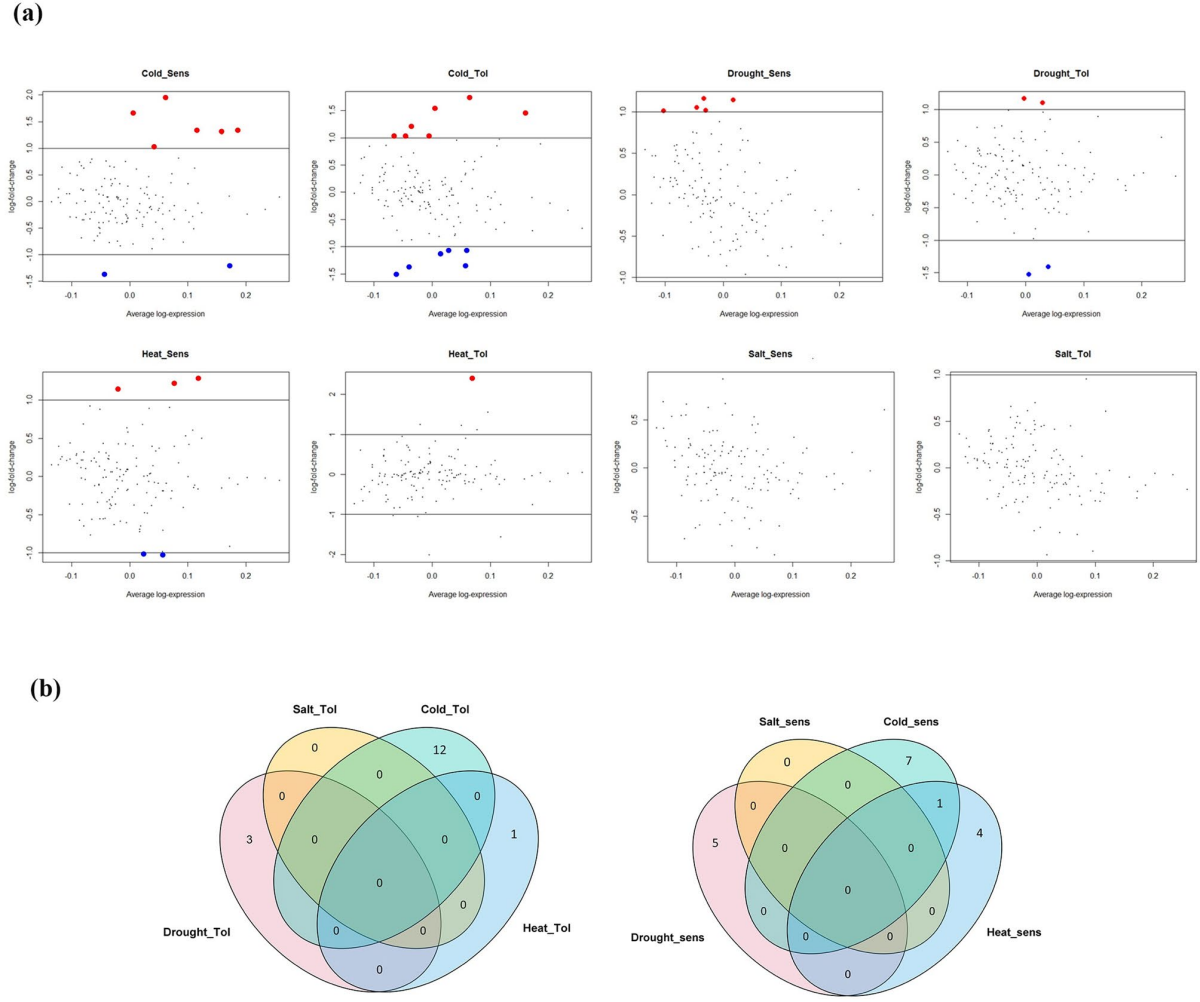
**a** Mean-difference (MD) plots showing differentially expressed genes under different stress conditions in rice genotypes at | log₂FC | >1 and adjusted *p*-value < 0.05. Red dots represent up-regulated DEGs, and blue dots represent down-regulated DEGs. **b** Venn diagrams illustrating the classification of genes induced by cold, drought, salinity, and heat stresses in sensitive and resistant genotypes

**Fig. 2:**
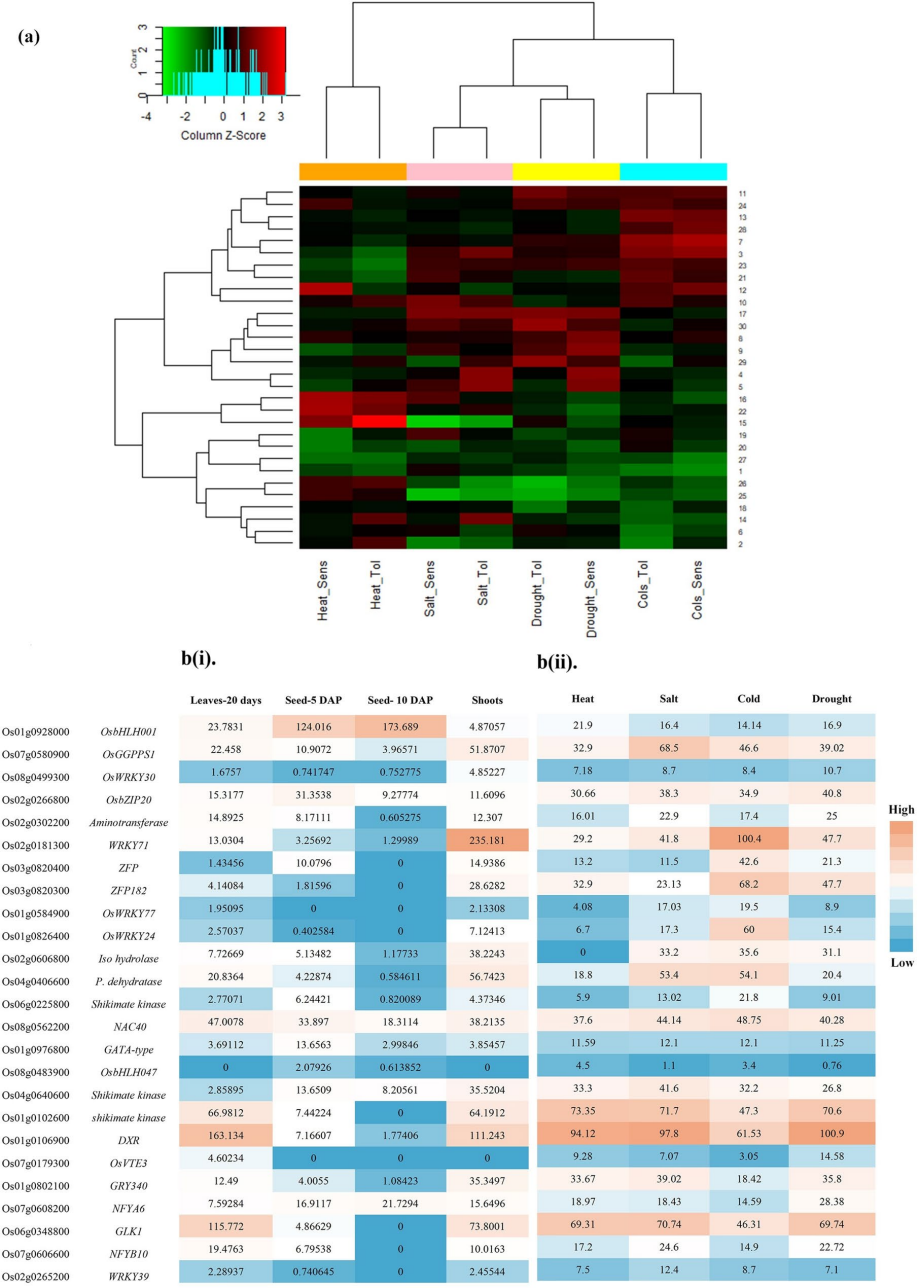
**a** Heatmap analysis of DEGs involved in tocopherol biosynthesis in response to abiotic stresses. Red colours represent highly expressed genes, and green colours represent lowly expressed genes under stress conditions relative to normal conditions. The genes could be grouped into three clusters. **bi** Expression analysis of DEGs using RNA-Seq for leaves (20-day-old), shoots and seedlings. **bii** heat, cold, salinity and drought stress. “red = up, blue = down”

The results of gene expression related to vitamin E biosynthesis in rice lines Azucena, DK151, IRAT109, Dagad deshi, Nonabokra, Li-Jiang-Xin-Tuan-Hei-Gu, K354, Annapurna (tolerant genotypes), and IR64, MI48, Zhonghua 11 (sensitive genotypes) subjected to drought, salinity, cold and heat stresses are briefly presented in Table 1. A set of 30 DEGs was identified under all stress conditions (Table 2). Comparatively, more DEGs responded to cold stress (19) than to other stress treatments (drought: 6, salinity: 0, heat: 5, Table 3). Stress from cold, drought, salinity, and heat resulted in the up-regulation of 12, 5, 0, and 4 Genes, and the down-regulation of 7, 1, 0, and 1 genes in sensitive and tolerant genotypes, respectively. Mean-Difference (MD) plots illustrating differentially expressed probes under drought, salinity, cold and heat stress are presented in Fig. 1a. Up-regulated DEGs are depicted using red dots, while blue dots represent down-regulated DEGs. Induced genes across all stresses were classified into regulatory and functional groups. The largest number of DEGs were transcription factors (TFs) from the bHLH, WRKY, bZIP and C2H2 families. We found 17 (2 down and 15 up-regulated) and 13 (six up and seven down-regulated) DEGs encoding TFs and functional genes, respectively, across all stress conditions in this study (Tables 2 and 3). In lines sensitive to drought stress, TFs such as *bHLH001* (*Os01g0928000*), *OsWRKY30* (*Os08g0499300*), *OsbZIP20* (*Os02g0266800*) were upregulated. Under cold stress, *WRKY71* (*Os02g0181300*), *ZFP15* (*Os03g0820400*), *ZFP182* (*Os03g0820300*), *OsWRKY77* (*Os01g0584900*), *OsWRKY24* (*Os01g0826400*) were upregulated in sensitive lines, and *NAC40* (*Os08g0562200*) was upregulated in tolerant lines. *WRKY71*, *ZFP15* and *OsWRKY24* were common between tolerant and sensitive lines under cold stress. Furthermore, under heat stress *OsWRKY77* (Os01g0584900), *GATA10* (Os01g0976800) and *OsbHLH047* (Os08g0483900) were upregulated. The functional genes that were upregulated in the different stresses were *OsGGPPS1* (Os07g0580900) and *aminotransferase* (Os02g0302200) under drought, *Isochorismatase hydrolase* (Os02g0606800), *Prephenate dehydratase* (Os04g0406600) and *Shikimate kinase* (Os06g0225800) under cold stress and Os04g0640600 under heat stress (Table 3). By comparing gene expression levels, the heat map of all DEGs revealed that DEGs showed similar expression patterns in sensitive and tolerant genotypes (Fig. 2a, Table S2), indicating that the primary response mechanism of stress was similar in both genotypes. We only observed a relatively specific pattern under cold stress, where two clusters with high expression (red) and low expression (green) can be identified. The Venn diagram describing specific and overlapping DEGs under the different stress treatments is shown in Fig. 1b. The Venn diagram demonstrates the number of unique up- and down-regulated genes at each stress in tolerant and sensitive genotypes. Most DEGs were uniquely associated with a specific stress (Fig. 1b), and only one gene was common between cold and heat in sensitive genotypes and another gene between drought and cold in tolerant genotypes.

**Table 2:** Differentially expressed genes (DEGs) characterized in response to different abiotic stresses

Accession	Name	Description
*Os01g0928000*	*OsbHLH001*	Similar to TF ICE1 (Inducer of CBF expression 1) (Basic helix- loop-helix protein 116) (*bHLH116*) (*AtbHLH116*)
*Os07g0580900*	*OsGGPPS1*	Similar to GGDP synthase
*Os08g0499300*	*OsWRKY30*	*WRKY30*
*Os02g0266800*	*OsbZIP20*	Transcriptional activator protein
*Os01g0102600*	*OsSK4*	Shikimate kinase domain containing protein
*Os02g0302200*	*Os02g0302200*	Similar to aminotransferase family protein
*Os01g0106900*	*OsDXR*	Similar to 1-deoxy-D-xylulose 5-phosphate reductoisomerase (Fragment)
*Os03g0820400*	*ZFP15*	Similar to *ZPT2*-*13*
*Os03g0820300*	*ZFP182*	TFIIIA-type zinc finger protein, Transcription activator, Abiotic stress tolerance
*Os01g0584900*	*OsWRKY77*	WRKY transcription factor 28-like (*WRKY5*) (*WRKY77*)
*Os01g0826400*	*OsWRKY24*	*WRKY24*
*Os07g0179300*	*OsVTE3*	Methyltransferase type 11 domain containing protein
*Os02g0181300*	*WRKY71*	WRKY transcription factor, Defense response
*Os01g0802100*	*OsIspE*	4-diphosphocytidyl-2-C-methyl-D-erythritol kinase, Isoprenoid biosynthesis, Chloroplast development
*Os02g0181300*	*WRKY71*	*WRKY* TF, Defense response
*Os07g0608200*	*OsHAP2G*	Similar to CCAAT-binding TF subunit B (CBF-B) (NF-Y protein chain A) (NF-YA) (CAAT-box DNA binding protein subunit A). Splice isoform Short
*Os03g0820400*	*ZFP15*	Similar to *ZPT2*-*13*
*Os02g0606800*	*OsNIC*	Isochorismatase hydrolase family protein
*Os01g0826400*	*OsWRKY24*	*WRKY24*
*Os06g0348800*	*OsGLK1*	Transfactor-like protein
*Os07g0606600*	*OsHAP3F*	Similar to nuclear factor Y transcription factor subunit B homolog
*Os04g0406600*	*Os04g0406600*	Prephenate dehydratase domain containing protein
*Os06g0225800*	*OsSK2*	Shikimate kinase domain containing protein
*Os08g0562200*	*OsNTL5*	Membrane-bound NAC-like transcription factor, Transcriptional repressor, Suppression of flowering
*Os02g0302200*	*Os02g0302200*	Similar to aminotransferase family protein
*Os01g0584900*	*OsWRKY77*	WRKY transcription factor 28-like (WRKY5) (WRKY77)
*Os01g0976800*	*OsGATA10*	Zinc finger, NHR/GATA-type domain containing protein
*Os02g0265200*	*OsWRKY39*	WRKY39
*Os08g0483900*	*OsbHLH047*	Helix-loop-helix DNA-binding domain containing protein
*Os04g0640600*	*OsSK3*	Shikimate kinase

**Table 3:** Up and down-regulated genes identified in this study

Stress	Line Details	Gene name	Accession	Location	Strand
Drought	Sensitive	*OsbHLH001*	*Os01g0928000*	Chr01: 40,714,216…40,717,112	-
		*OsGGPPS1*	*Os07g0580900*	Chr07: 23,506,852…23,509,287	-
		*OsWRKY30*	*Os08g0499300*	Chr08: 24,645,932…24,649,832	-
		*OsbZIP20*	*Os02g0266800*	Chr02: 9,535,430…9,538,420	-
	resistant	aminotransferase	*Os02g0302200*	Chr02: 11,716,855…11,736,527	+
Cold	Sensitive	*WRKY71*	*Os02g0181300*	Chr02: 4,544,306…4,544,841	+
		*ZFP15*	*Os03g0820400*	Chr03: 34,427,704…34,428,391	+
		*ZFP182*	*Os03g0820300*	Chr03: 34,424,180…34,425,030	+
		*OsWRKY77*	*Os01g0584900*	Chr01: 22,731,943…22,733,237	+
		*OsWRKY24*	*Os01g0826400*	Chr01: 35,347,982…35,350,546	+
	resistant	*WRKY71*	*Os02g0181300*	Chr02: 4,544,306…4,544,841	+
		*ZFP15*	*Os03g0820400*	Chr03: 34,427,704…34,428,391	+
		Isochorismatase hydrolase	*Os02g0606800*	Chr02: 23,773,136…23,775,628	+
		*OsWRKY24*	*Os01g0826400*	Chr01: 35,347,982…35,350,546	+
		Prephenate dehydratase	*Os04g0406600*	Chr04: 20,169,585…20,171,246	+
		Shikimate kinase	*Os06g0225800*	Chr06: 6,495,002…6,497,904	-
		*NAC40*	*Os08g0562200*	Chr08: 28,144,876…28,149,227	-
Heat	Sensitive	*OsWRKY77*	*Os01g0584900*	Chr01: 22,731,943…22,733,237	+
		*OsGATA10*	*Os01g0976800*	Chr01: 43,172,242…43,173,072	-
		*OsbHLH047*	*Os08g0483900*	Chr08: 23,894,602…23,896,537	-
	resistant	Shikimate kinase	*Os04g0640600*	Chr04: 32,582,187…32,585,691	-
Down-regulated genes					
Drought	resistant	Shikimate kinase	*Os01g0102600*	Chr01: 145,603…147,847	+
Cold	Sensitive	*OsDXR*	*Os01g0106900*	Chr01: 374,728…380,713	-
		*OsVTE3*	*Os07g0179300*	Chr07: 4,182,044…4,184,048	-
	resistant	*OsIspE*	*Os01g0802100*	Chr01: 33,971,226…33,976,464	+
		*OsHAP2G*	*Os07g0608200*	Chr07: 24,996,740…25,001,214	-
		*OsGLK1*	*Os06g0348800*	Chr06: 14,078,261…14,082,203	+
		*OsHAP3F*	*Os07g0606600*	Chr07: 24,921,067…24,921,998	-
		aminotransferase	*Os02g0302200*	Chr02: 11,716,855…11,736,527	+
Heat	Sensitive	*OsWRKY39*	*Os02g0265200*	Chr02: 9,446,779…9,448,835	+

### In Silico RNA-seq expression analysis of DEGs

Expression data of DEGs were obtained from RNA-Seq assays for leaves (20-day-old), shoots, seedlings and from the Plant Public RNA-seq Database for drought, salinity, heat and cold stresses. Among the identified genes, *OsbHLH001* (*Os01g0928000*), *OsGGPPS1* (*Os07g0580900*), *OsbZIP20* (*Os02g0266800*), *WRKY71* (*Os02g0181300*), *Isochorismatase hydrolase* (*Os02g0606800*), *Prephenate dehydratase* (*Os04g0406600*), *NAC40* (*Os08g0562200*), *Shikimate kinase* (*Os04g0640600*), *DXR* (*Os01g0106900*) and *GLK1* (*Os06g0348800*) were associated with tocopherol content and also showed a higher expression in different rice tissues. The highest expression was found for *WRKY71*, which was expressed in shoots of IR64 under cold stress. *DXR* and *GLK1* were expressed in all tissues. Shikimate kinase and *GLK1* were highly expressed in seedlings (Fig. 2b(i)). *OsGGPPS1*, *OsbZIP20*, *WRKY71*, *NAC40*, *shikimate kinase*, *DXR*, *GLK1* were expressed in all stresses (Fig. 2 (ii)). *ZFP182* was expressed in experiments involving heat, cold, and drought stress. *GRY340* was expressed in heat, salinity and drought stress. *Isochorismatase hydrolase* and *Prephenate dehydratase* were expressed under salinity and cold stress. Additionally, *Isochorismatase hydrolase* was expressed under drought stress conditions. *ZFP15* and *OsWRKY24* were specifically expressed in response to cold stress.

### Analysis of Co-expressed genes in PPI networks

**Fig. 3:**
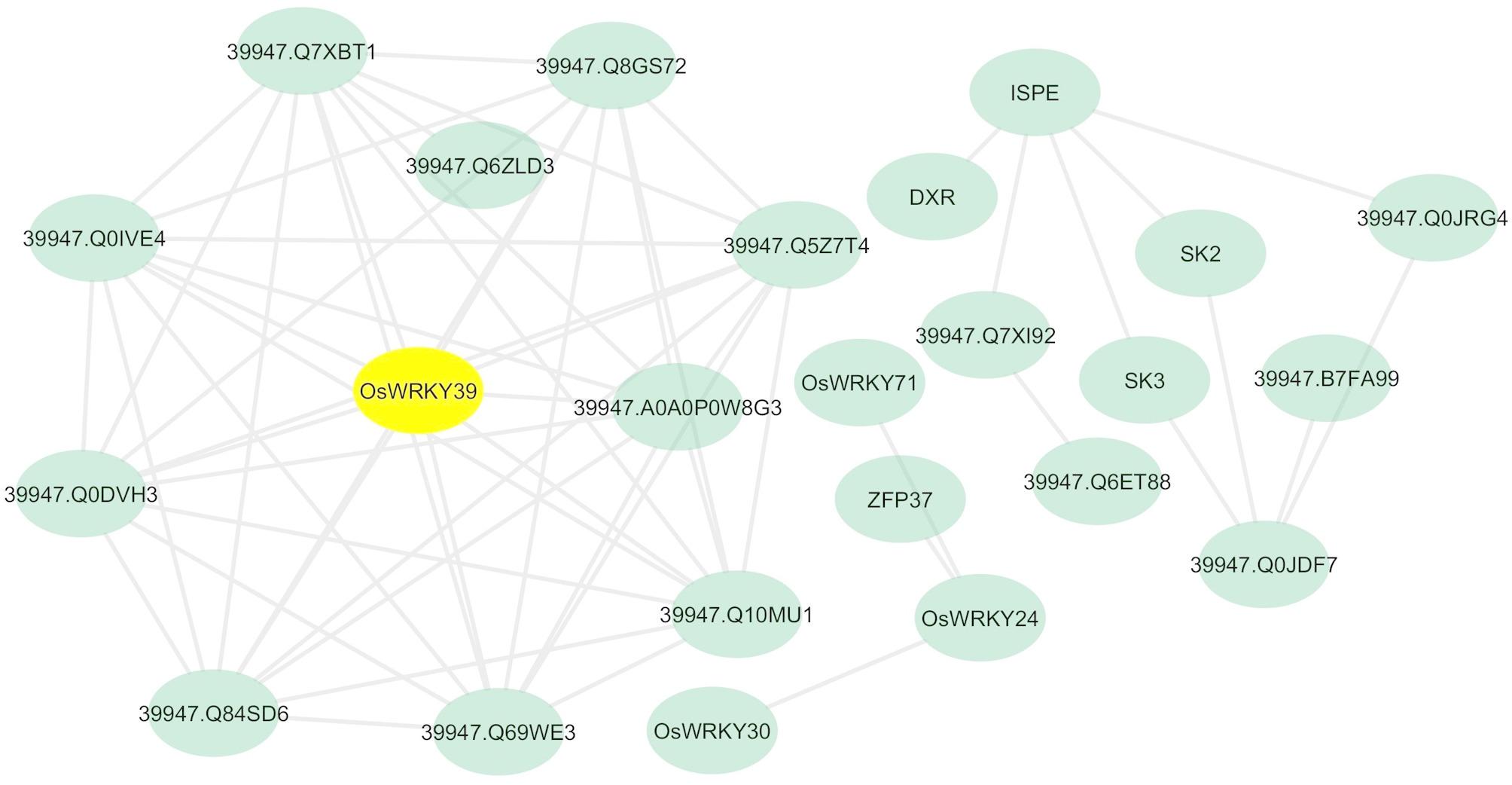
Interactions of responsive genes to vitamin E biosynthesis in rice. Each node represents a protein, and each edge represents an interaction. The web-based tool “String” (http://string-db.org/) was used to predict the interactions

To understand the core stress-response signalling and metabolic changes, we performed a PPI networks investigation. The stress-responsive genes were used as ‘seeds’ to search the protein-protein interaction networks database (Fig. 3). A total of 60 interactions between 30 DEGs were identified. From our analysis, we observed three clusters. The PPI network analysis revealed that co-expressed genes are involved in the biosynthesis and metabolism of chorismate and shikimate, as well as in the negative regulation of signalling pathways, the biosynthesis of aromatic amino acids, cellular amino acid metabolism, and the metabolic processes of small molecules. The most significant nodes in co-expressed genes networks were *OsWRKY30* (Os08g0499300), *OsWRKY24* (Os01g0826400), *WRKY71* (Os02g0181300), *ZFP15* (Os03g0820400), *OsWRKY39* (Os02g0265200), *aminotransferase* (Os02g0302200**)**, *Prephenate dehydratase* (Os04g0406600), *OsSK4* (Os01g0102600**)**, *OsSK2* (Os06g0225800), *OsSK3* (Os04g0640600), *OsIspE* (Os01g0802100) and *DXR* (Os01g0106900). Based on the PPI network constructed from the DEGs, one hub gene, *OsWRKY39*, was identified and shown to be a newly identified candidate gene involved in the response to stress conditions in rice.

### Gene ontology and KEGG pathway

**Fig. 4:**
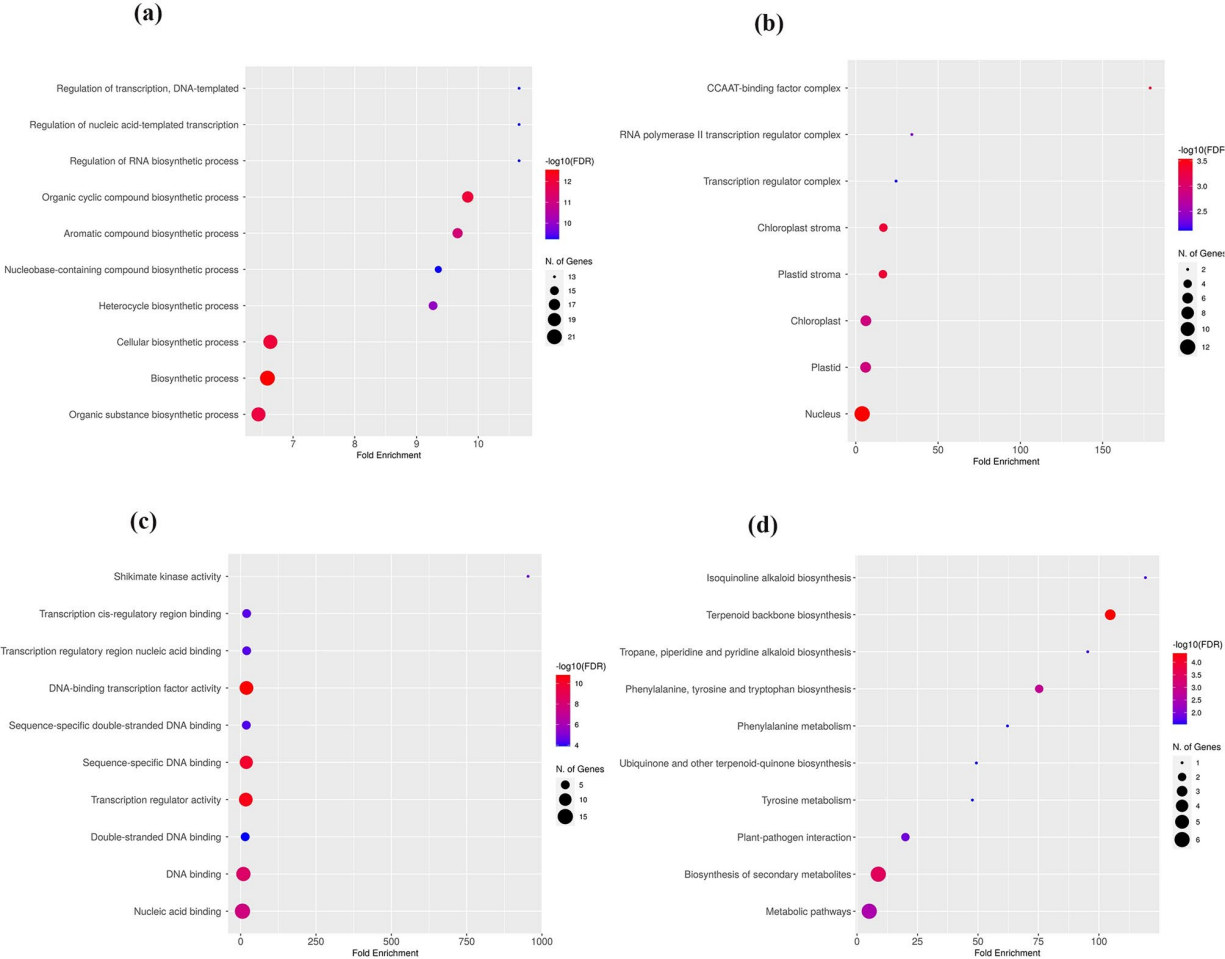
Functional enrichment analysis of candidate genes. GO analysis revealed that DEGs were significantly enriched in **(a)** biological process, **(b)** cellular component **(c)** molecular function **(d)** significantly enriched KEGG terms obtained from KEGG analysis. *KEGG* Kyoto Encyclopedia of Genes and Genomes, *GO* Gene Ontology

The enrichment analysis highlighted proteins involved in biological processes, cellular components, molecular function or KEGG pathways. The gene ontology analysis revealed that vitamin E-related genes were preferentially involved in biological processes, including regulation of transcription, DNA-template, regulation of nucleic acid-template transcription, regulation of RNA biosynthetic process, organic cyclic compound biosynthetic process, aromatic compound biosynthetic process, heterocycle biosynthetic process, biosynthetic process and organic substance biosynthetic process (Fig. 4a). The GO analysis further showed that the DEGs exhibited prominent enrichment in several categories, including the CCAAT-binding factor complex, RNA polymerase II transcription regulator complex, chloroplast stroma, plastid stroma, chloroplast, plastid and nucleus (Fig. 4b). The molecular functions of the candidate genes included shikimate kinase activity, transcription cis-regulatory region binding, DNA binding and nucleic acid binding (Fig. 4c). Finally, the KEGG pathway analysis demonstrated that DEGs were significantly enriched for isoquinoline alkaloid biosynthesis, terpenoid backbone biosynthesis, tropane, piperidine and pyridine alkaloid biosynthesis, phenylalanine, tyrosine and tryptophan biosynthesis, phenylalanine metabolism, ubiquinone and other terpenoid-quinone biosynthesis, tyrosine metabolism, biosynthesis of secondary metabolites, metabolic pathways (Fig. 4d). The aromatic amino acids (AAA), phenylalanine (Phe), tyrosine (Tyr) and tryptophan (Trp) are synthesised via the shikimate pathway and the subsequent branched aromatic amino acid metabolic pathway, with chorismate serving as a major branch point for the intermediate metabolites. In this way, Tyr serves as a precursor of several families of secondary metabolites, including vitamin E [38].

**Fig. 5:**
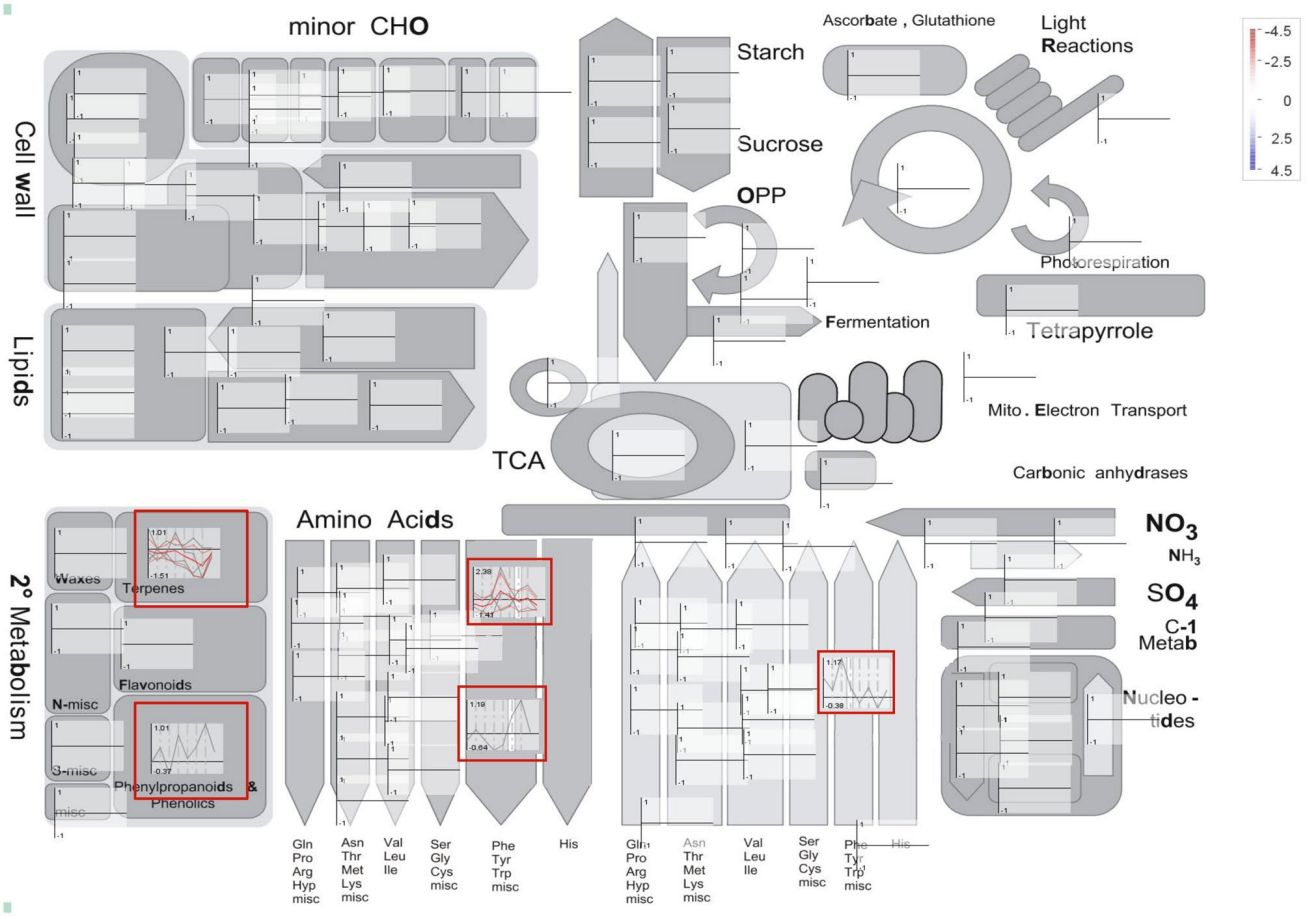
MapMan analysis - secondary metabolism overview associated with DEGs. The main genes effective in the biosynthesis of terpenes and aromatic amino acids, which are the precursors of tocopherol biosynthesis. A conventional red-to-blue scale indicated up-regulation (red) or down-regulation (blue)

To investigate the significant metabolic pathways in which DEGs are involved, we analysed the metabolism overview associated with the 30 DEGs (Fig. 5). MapMan analysis of rice genes associated with vitamin E synthesis indicates that DEGs are mainly effective in the biosynthesis of terpenes and aromatic amino acids, which serve as precursors in the biosynthesis of tocopherol. These results imply that rice plants trigger the vitamin E pathways as part of their stress response.

## Discussion

The changing global climate has given rise to abiotic stresses that adversely affect the metabolic activities of plants, Limiting their growth and agricultural output, and posing a serious threat to food production. The statistics suggest that various abiotic stressors can reduce the agricultural output by as much as 50% [39, 40]. Abiotic stresses commonly lead to the production of reactive oxygen species (ROS), which results in cellular oxidation. Throughout evolution, plants have devised efficient enzymatic and non-enzymatic antioxidant strategies to counteract the harmful effects of ROS. Among the emerging non-enzymatic anti-oxidative technologies, the chloroplast lipophilic antioxidant vitamin E shows excellent promise. Biosynthesis and aggregation of tocopherol have been considered major host plant responses to cope with the harmful effects of oxidative stress [41]. Tocopherols play key roles in regulating a stable cellular redox condition and in their natural antioxidant defence systems. These are considered a primary conserved system to impart resistance against stress in plants. The key role of tocopherols as an antioxidant involves firmly holding the phospholipid bilayer of polyunsaturated fatty acyl chains [42]. Tocopherol-deficient *vte2* mutants in *Arabidopsis thaliana* and *Oryza sativa* have both been shown to be susceptible to cold stress despite having strikingly different phenotypes when grown under ideal growth conditions [43, 44].

**Fig. 6:**
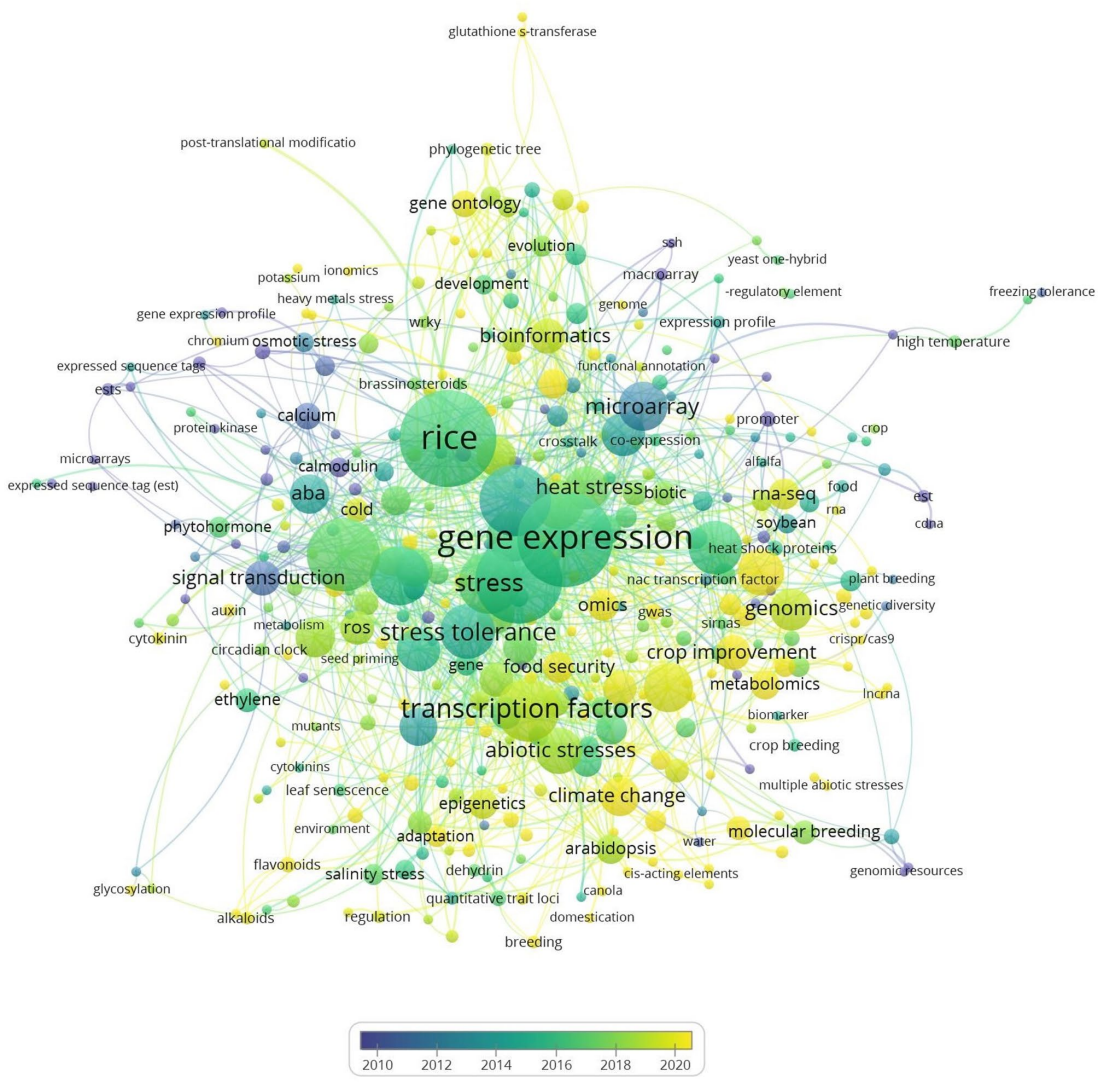
Bibliometric map of keywords used in this article from 2010 to 2023. Data were collected from the ScienceDirect database using the keywords search: “cold stress, drought stress, heat stress, microarray, salinity stress and rice”

To gain a comprehensive understanding of studies on abiotic stresses in rice, a co-occurrence analysis was performed using high-frequency keywords to construct a knowledge map. Bibliometric networks were visualised using scientific papers published between the years 2010 and 2023 [45] (Fig. 6). The results show that in recent years, the primary focus has been on techniques and methods for analysing transcriptomes to develop a better understanding of plant responses to abiotic stress. Additionally, an increasing emphasis on regulatory elements, such as transcription factors, underscores the necessity for further research. As Fig. 6 shows, even though high-throughput techniques are now being implemented, our understanding of abiotic signalling cascades is not yet well advanced, neither in general nor for specific antioxidant stress responses, such as vitamin E.

This study primarily relied on genes identified in a previous GWAS conducted by our group [33, 34], providing a strong scientific foundation. We aimed to investigate the effects and behaviour of these genes—mainly transcription factors—under various stress conditions in rice. To date, no comprehensive study has been conducted on transcription factors potentially involved in vitamin E biosynthesis under different stress conditions in rice. Therefore, this study is unique in this regard.

In this study, we analysed microarray data from drought, cold, heat, and salinity stress experiments in tolerant and sensitive rice Genotypes. The dataset included 136 probe sets derived from leaves, flag leaves, and seedlings. In leaves, vitamin E is primarily located in chloroplast membranes, plastoglobules, and thylakoid membranes, placing it near the photosynthetic apparatus. Consequently, tocopherols are considered essential antioxidants that protect photosynthetic structures from ROS generated during photosynthesis.

Seedlings experience severe defects during germination and early growth due to the accumulation of non-enzymatic lipid peroxides and hydroxy fatty acids. This highlights the critical role of tocopherols in preventing lipid oxidation during early developmental stages [46]. Across all stress conditions, a total of 30 differentially expressed genes (DEGs) were identified: 19 under cold, 6 under drought, 0 under salinity, and 5 under heat stress. Among them, we identified six structural genes (*OsGGPPS1*, *aminotransferase*, *isochorismatase hydrolase*, *prephenate dehydratase*, and *shikimate kinase*) and 12 transcription factors (*OsbHLH001*, *OsWRKY30*, *OsbZIP20*, *WRKY71*, *ZFP15*, *ZFP182*, *OsWRKY77*, *OsWRKY24*, *NAC40*, a zinc finger protein, *OsGATA10*, and *OsbHLH047*) that were upregulated under at least one environmental stress condition. These findings were supported by RNA-seq expression datasets, in which *OsGGPPS1*, *isochorismatase hydrolase*, *prephenate dehydratase*, *shikimate kinase*, *OsbZIP20*, *WRKY71*, *ZFP15*, *ZFP182*, *OsWRKY24*, and *NAC40* were upregulated in response to one or more stresses. The upregulated genes were primarily involved in aromatic compound biosynthesis, negative regulation of gibberellic acid signalling, shikimate metabolism, and chorismate biosynthesis. Conversely, *OsVTE3*, *OsDXR*, an aminotransferase, *HAP3F*, *OsGLK1*, *HAP2G*, and *GRY340* were downregulated under cold stress; shikimate kinase was downregulated under drought stress; and *OsWRKY39* was upregulated under heat stress. The shikimate pathway is a key metabolic route in plants and some microorganisms that produces the aromatic amino acids phenylalanine, tyrosine, and tryptophan. It also provides precursors for numerous secondary metabolites, most notably vitamin E [47]. MapMan is a valuable tool to provide global views of diverse aspects of data and can be used to functionally classify rice genes [48]. Characterised genes in metabolism analysis using the MapMan comprehensive overview highlighted the key pathways influenced by tocopherol biosynthesis. Our results underline the crucial role of vitamin E in protecting against oxidative stress. Most transcription factors involved in the tocopherol biosynthetic pathway were upregulated in response to abiotic stress, suggesting that the activation of these regulatory networks may contribute to stress tolerance in rice.

### Drought stress

Drought is one of the most severe abiotic stresses, responsible for reducing global crop yields by up to 45%. It results from a combination of reduced soil water availability and increased evaporation under dry climatic conditions. Drought stress impairs plant growth and yield through nutrient limitation, ion toxicity, and disruption of photosynthetic processes. *OsbHLH001*, a member of the basic helix-loop-helix (bHLH) TF family, is implicated in multiple physiological processes, including drought stress responses. While extensive studies in *A*. *thaliana* have elucidated the functional diversity of bHLH TFs, their specific roles in economically important crops, such as rice, remain less characterised [49]. *OsWRKY30*, a WRKY transcription factor, interacts with multiple mitogen-activated protein kinases (MAPKs), including *OsMPK3*, *OsMPK4*, *OsMPK7*, *OsMPK14*, *OsMPK20-4*, and *OsMPK20-5*. It is phosphorylated by *OsMPK3*, *OsMPK7* and *OsMPK14*, suggesting its role downstream of MAPK signalling pathways. Overexpression of *OsWRKY30* significantly enhances drought tolerance in rice, indicating its regulatory function in stress adaptation [50]. Similarly, *OsbZIP20*, a member of the bZIP transcription factor family, enhances drought and salt stress tolerance when overexpressed, likely through the upregulation of stress-responsive genes. This highlights the importance of bZIPs in abiotic stress signalling and gene regulation [51].

### Heat stress

Heat stress is another major abiotic factor that Limits crop productivity, particularly in the context of rising global temperatures. Estimates suggest that a 1 °C increase in temperature can reduce rice yields by 10% and wheat yields by 3–4% [52, 53]. Prolonged exposure to temperatures above 30 °C causes a measurable decline in yields of maize and barley [54]. Among the transcription factors involved in heat stress response, WRKYs are well-documented. OsWRKY77, in particular, binds to W-box elements in the promoters of downstream genes and modulates gene expression in response to high temperatures. These transcription factors integrate multiple signalling pathways, mediating both transcriptional reprogramming and hormonal responses to heat stress [55].

### Cold stress

Wang et al. (2023) demonstrated, through transcriptomic and QTL analyses, that *OsWRKY71* encodes a transcription factor active in the GA signal transduction process, which also enhances cold tolerance in plants [56]. *OsWRKY71* has also been shown, via transcriptomic studies and RNA gel blot analysis, to positively regulate cold tolerance by modulating downstream gene expression [57]. In addition, *ZFP182*, a zinc finger protein, enhances tolerance to cold, salt, and drought when overexpressed. Its role in promoting osmolyte accumulation, such as proline and soluble sugars, under stress underscores the importance of metabolic adjustments in stress resilience [58].

Contrastingly, *ZFP15*, although containing stress-responsive elements in its promoter region, does not show transcriptional induction under cold or drought conditions, indicating functional divergence among zinc finger proteins [59]. Other WRKY TFs, such as *OsWRKY24*, show transcriptional upregulation in response to cold, salicylic acid (SA), and methyl jasmonate (MeJA) [60]. Furthermore, NAC transcription factors, including *NAC40*, are induced by cold stress and play a crucial role in orchestrating cold-responsive transcriptional networks. These TFs regulate a wide array of downstream targets, contributing to enhanced tolerance and adaptability [61].

Interestingly, a greater number of genes and transcription factors related to the vitamin E biosynthesis pathway are upregulated under cold stress, whereas such upregulation is not observed under salt stress. Chaudhary and Khurana (2009) observed an increased transcript abundance of *HPPD*, the first committed enzyme involved in the biosynthesis of vitamin E, in rice during drought and cold stress, but not under salt stress conditions [62]. Similarly, Ji et al. (2016) reported that the expression of *HPPD* in sweet potato decreased during salt stress induced by NaCl [63]. This difference indicates that the regulation of the vitamin E biosynthesis pathway in rice is highly dependent on the type of stress. Cold stress primarily causes membrane rigidification and oxidative damage in chloroplasts, thereby increasing the demand for lipophilic antioxidants, such as tocopherols, to maintain membrane integrity and photosynthetic efficiency. In contrast, salt stress primarily induces osmotic and ionic stress, activating different signalling pathways that do not necessarily involve increased tocopherol biosynthesis.

A study by Luo et al. (2021) demonstrated that genes and transcription factors associated with the vitamin E biosynthesis pathway are specifically activated under cold stress conditions. This activation is triggered by calcium (Ca²⁺) signalling induced by the *COLD1* receptor in rice, leading to increased expression of genes such as *VTE4* (responsible for α-tocopherol, or vitamin E, biosynthesis) and *VKORC1* (a key gene in the vitamin K1 pathway) within chloroplasts. The elevated expression of these genes activates the biosynthesis pathways of vitamins E and K1, playing an important role in protecting chloroplasts from cold-induced damage. α-Tocopherol, as a lipophilic antioxidant, prevents oxidative damage and membrane rigidification in chloroplasts [64].

Under heat and drought stress, plants primarily face water deficit, osmotic changes, and hormonal regulation (e.g., abscisic acid signalling), which may result in a lower immediate requirement for upregulating vitamin E biosynthesis or activating alternative protective pathways. Moreover, signalling and gene regulation related to other antioxidant systems such as glutathione, ascorbic acid, and oxidative enzymes (e.g., superoxide dismutase, catalase) tend to be more prominent. This suggests that plants employ different protective mechanisms to cope with these stress types, reducing the necessity for increased expression of vitamin E biosynthesis genes.

Our study on rice reveals that the differential expression of transcription factors (TFs) plays a crucial role in enhancing transcriptional regulation across multiple pathways, thereby improving stress tolerance through increased vitamin E biosynthesis. However, to fully understand the regulatory roles of these TFs in vitamin E biosynthesis under stress conditions, further functional characterisation using approaches such as gene knockout or overexpression in rice is needed.

Previous studies have demonstrated that plant responses to combined or sequential stresses are unique and cannot be inferred from single-stress responses, largely due to the complex interactions among signalling pathways [65–67]. Consistent with this, Anwar et al. (2025) reported that transcriptional responses in rice are highly context-dependent, exhibiting distinct expression patterns under individual, combined, and sequential stresses [68]. In this study, we investigated plant responses to stress using transcriptomics data. Although this approach provides valuable insights, it is limited to a single-omics layer. Contemporary research increasingly emphasises the importance of integrated panomics approaches to achieve a more comprehensive understanding of plant responses to complex stresses. Addressing this limitation in future studies through the integration of multi-omics will be crucial for a more holistic understanding of plant responses to complex stress conditions.

## Conclusions

Here, we used 231 microarray samples exposed to drought, cold, heat or salinity stress and related the results to the biosynthesis of vitamin E, identifying 30 DEGs using a p-value of < 0.05 and |log_2_FC| > 1. To follow up on these observations, we performed in silico expression analysis of the DEGs and a PPI network analysis using bioinformatics tools. Our findings show that 13 structural Genes and 17 transcription factors are involved in the biosynthesis of vitamin E in response to drought, cold, and heat stresses in rice. The microarray data analyses show that abiotic stresses induce genes involved in vitamin E biosynthesis, except for the salinity stress. Notably, vitamin E production is being induced in response to stress, more specifically in response to cold. Overall, our results provide new insights into how vitamin E-related genes respond to stress in rice. Future studies should include qRT-PCR validation of key DEGs under salinity and cold stress to confirm expression trends. Additionally, functional characterisation of candidate transcription factors through gene knockout or overexpression in rice could clarify their regulatory roles in vitamin E biosynthesis under stress conditions.

## Supplementary Information

Supplementary Material 1.

## Data Availability

Data is provided within the manuscript or supplementary information files.
